# Q-ing tumor glutaminase therapy

**DOI:** 10.18632/oncotarget.6301

**Published:** 2015-11-09

**Authors:** Zachary E. Stine, Chi V. Dang

**Affiliations:** Abramson Family Cancer Research Institute, Abramson Cancer Center, University of Pennsylvania Perelman School of Medicine, Philadelphia, PA, USA

**Keywords:** cancer, metabolism, glutaminase

Cancer cells reprogram metabolism to provide the energy, nucleotides, lipids, amino acids and other building blocks required to proliferate and survive in the stressful tumor microenvironment [1]. Changes in cancer metabolism were first noted in the 1920's with Otto Warburg's discovery that tumors show increased aerobic conversion of glucose to lactate (termed the Warburg Effect). A recent revival of interest in cancer metabolism has been fueled by the discovery that hypoxia and the constitutive activation of MYC, KRAS, PI3K/AKT/mTOR signaling and other pathways drives the rewiring of tumor metabolism by controlling the expression and activity of key metabolic enzymes. The sustained proliferation driven by oncogene activation and loss of checkpoints leaves tumors ‘addicted’ to reprogrammed metabolism, opening a therapeutic window.

Glutamine, the most abundant amino acid in the blood, can be taken up by transporters (including SLC1A5/ASCT2) and utilized as a source of carbon and nitrogen for energy production and biosynthesis [2]. Glutamine can be converted to glutamate by the enzyme glutaminase, which, in humans, is encoded by the genes *GLS* (kidneytype glutaminase) and *GLS2* (liver-type glutaminase). Glutamate can then be converted to the tricarboxylic acid (TCA) cycle intermediate α-ketoglutarate by glutamate dehydrogenase or transaminases. While glucose-derived pyruvate is shunted away from the mitochondria in tumors, glutamine-derived α-ketoglutarate can fuel the TCA cycle. Although Warburg's observations led to the assumption that the TCA cycle was not critical in tumors, mitochondrial TCA metabolism provides important precursors for the synthesis of cellular building blocks [1, 2].

MYC, one of the most frequently deregulated genes in cancer, is a transcription factor that promotes cell proliferation, induces the Warburg effect, and drives ribosome biosynthesis and translation to increase cell mass [3]. MYC-driven cell transformation induces dependence on extracellular glutamine and upregulates expression of SLC1A5 and GLS [1–4].

As GLS is broadly expressed in many cancer types and catalyzes the first step of glutamine catabolism, it represents a potential anti-cancer therapy target. While initial attempts to target glutamine metabolism with glutamine analogs led to wide spread toxicity, the development of an allosteric GLS inhibitor (BPTES, bis-2-(5-phenylacetamido-1,2,4-thiadiazol-2-yl)ethyl sulfide) showed promise *in vitro* and in xenografts models [5]. Recently, we published a study testing the ability of GLS inhibition to treat a genetically engineered mouse model of MYC-driven hepatocellular carcinoma (HCC), termed the LAP/MYC model [6]. We found that LAP/MYC HCC tumors showed increased *Gls* expression and decreased *Gls2* expression compared to surrounding tissue, and confirmed that the upregulation of *GLS* and downregulation of *GLS2* is also found in human HCC. We showed that treatment with BPTES, specific to the GLS isoform, prolonged survival of LAP/MYC mice compared to vehicle treated controls. BPTES-treated mice showed smaller tumors with decreased staining of the proliferation marker KI-67. Consistent with GLS inhibition, tumors treated with BPTES showed increased glutamine levels and decreased glutamate levels compared to controls. BPTES treatment was well tolerated in mice. Then, using a MYC-driven cell line as a model to study the effects of GLS inhibition, we demonstrated that BPTES treatment blocked DNA replication, resulting in cell death. Further, we confirmed the *in vivo* specificity of BPTES by rescuing xenograft growth with the expression of a BPTES resistant GLS mutant.

**Figure 1:**
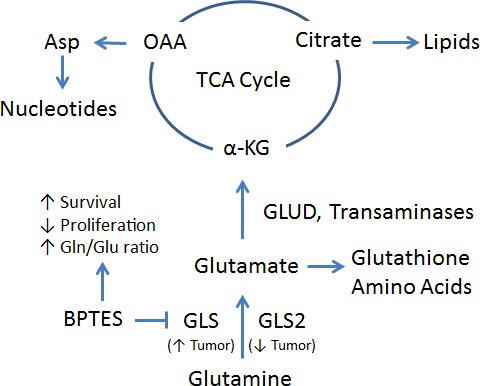
Glutamine (Gln) is converted to glutamate (Glu) by glutaminase, encoded for by *GLS* (upregulated in tumor) and *GLS2* (downregulated in tumor) In addition to its role in glutathione and amino acid synthesis, glutamate can then be converted to α-Ketoglutarate (α-KG) by glutamate dehydrogenase (GLUD) or aminotransferases. The TCA cycle provides citrate for lipid synthesis and oxaloacetate (OAA), which can be converted to the nucleotide synthesis precursor aspartate (Asp). BPTES inhibits GLS to block the conversion of glutamine to glutamate and prolong survival in the LAP/MYC model.

While BPTES shows encouraging preclinical efficacy, a BPTES related compound (CB-839) with improved pharmacological properties has entered phase I clinical trials [7]. Many challenges and opportunities remain as GLS inhibition enters the clinic, including the need to identify tumors that may respond to GLS inhibition. While *in vitro* studies show that cell lines of many cancer types depend on glutamine and GLS activity, some recent studies indicate that tumors may not be as commonly glutamine dependent as cells grown in a dish [2]. However, these studies have been limited in scope and will require further examination.

Prediction of therapeutic response to GLS inhibition will require the identification of biomarkers, development of new tools, and a detailed understanding of how mutational status interacts with the tissue type of origin to control tumor metabolism. While MYC has been shown to induce glutamine dependence *in vitro* and reprogram glutamine metabolism in various transgenic models *in vivo*, the tumor tissue of origin can impact how glutamine metabolism is affected by MYC expression. For example, while transgenic MYC expression in the LAP/MYC model reprograms glutamine metabolism and promotes glutaminase dependence, a MYC-driven lung tumor model does not exhibit reprogrammed glutamine metabolism and shows increased expression of glutamine synthetase [4]. Studies suggest that potential predictors of response to GLS inhibition include high expression of the GLS splice isoform GAC, low glutamine to glutamate ratio and low expression of genes that may circumvent the requirement for GLS activity, such as Pyruvate Carboxylase and GLS2 [2, 7]. Similar to the use of 18F-fluorodeoxyglucose Positron Emission Tomography (FDG-PET) to image tumors through their avid uptake of glucose, fluorinated glutamine probes have been developed and are in clinical trials [2]. It remains to be seen if high tumor 18F-glutamine uptake predicts therapeutic response.

Glutamine metabolism plays a diverse role in metabolism, controlling cellular energetics, redox state, amino acid production, cell signaling and nucleotide synthesis. The centrality of GLS in these diverse cellular functions makes GLS inhibition an ideal candidate for combination therapies. In addition to reports already in the literature of GLS showing promise in combination therapy in preclinical studies, we speculate that GLS inhibition will show synthetic lethality with drugs that perturb cellular metabolism, nucleotide synthesis, redox state or DNA repair among others.
